# Imperfect integration: Congruency between multiple sensory sources modulates decision-making processes

**DOI:** 10.3758/s13414-021-02434-7

**Published:** 2022-04-22

**Authors:** Dominik Krzemiński, Jiaxiang Zhang

**Affiliations:** 1 Cardiff University Brain Research Imaging Centre, School of Psychology, Cardiff University, Cardiff, UK; 2 Department of Zoology, University of Cambridge, Cambridge, UK

**Keywords:** Decision-making, Multiple sources, Attention, Speed–accuracy trade-off, Cognitive model, Neural-mass model

## Abstract

Decision-making on the basis of multiple information sources is common. However, to what extent such decisions differ from those with a single source remains unclear. We combined cognitive modelling and neural-mass modelling to characterise the neurocognitive process underlying perceptual decision-making with single or double information sources. Ninety-four human participants performed binary decisions to discriminate the coherent motion direction averaged across two independent apertures. Regardless of the angular distance of the apertures, separating motion information into two apertures resulted in a reduction in accuracy. Our cognitive and neural-mass modelling results are consistent with the hypotheses that the addition of the second information source led to a lower signal-to-noise ratio of evidence accumulation with two congruent information sources, and a change in the decision strategy of speed–accuracy trade-off with two incongruent sources. Thus, our findings support a robust behavioural change in relation to multiple information sources, which have congruency-dependent impacts on selective decision-making subcomponents.

**Supplementary Information:**

The online version contains supplementary material available at 10.3758/s13414-021-02434-7.

## Introduction

Making rapid decisions on the basis of noisy information is a hallmark of voluntary behaviour. For choices made within a range of 1–2 s, converging evidence from humans (Heekeren, Marrett, Bandettini, & Ungerleider, 2004) and non-human primates (Roitman & Shadlen, 2002) have supported an evidence accumulation framework governing decision-making from perceptual (Shadlen & Newsome, 2001), value (Zajkowski, Krzemiński, Barone, Evans, & Zhang, 2021), or memory (Ratcliff, 1978) information: the information is integrated over time until the accumulated information in favour of one option reaches a response threshold (but see also Cisek, Puskas, and El-Murr (2009) for an alternative account). This integration process reduces the noise in the accumulated information and facilitates optimal behaviour in terms of accuracy and speed (Bogacz, 2007). A large family of sequential sampling models (Bogacz, Brown, Moehlis, Holmes, & Cohen, 2006) can describe adequately the cognitive (Ratcliff & McKoon, 2008) and neural (Wang, 2002) processes during evidence accumulation.

Much of the research to date on simple visual perceptual decisions has focused on evidence accumulation from a single information source (Gold & Shadlen, 2007). Understanding how a decision-maker integrates information from multiple sources is equally important. In preferential decisions with multiple attributes, such as buying a car based on its colour and price, sequential sampling models can effectively account for various biases and heuristics (Busemeyer, Gluth, Rieskamp, & Turner, 2019), supporting evidence accumulation as a parsimonious decision-making framework for distributed information sources.

Another common scenario exists: making decisions by integrating the *same* type of information originated from multiple sources. For example, when approaching a T junction, a car driver has to consider incoming traffic from both left and right sides of the main road; while entering a roundabout, the driver only needs to attend to one side because all vehicles circulate in one direction. An intriguing issue is: how does the presence of multiple information sources affect behavioural performance. Research on visual search provide circumstantial evidence to imply an imperfect integration of multiple sources, because of the limited capacity of the attentional system. When locating a target among similar distractors or filtering out task irrelevant information, there is a robust behavioural (Palmer, 1995) and neural (Reynolds & Chelazzi, 2004) cost in relation to selective attention on multiple sources. However, two important questions are yet to be addressed. First, when the total amount of information remains unchanged, does separating information into multiple congruent and incongruent sources have the same impact on behaviour? Second, does making decisions with additional information sources lead to a change in the speed of evidence accumulation, the decision threshold, or both?

**Fig. 1:**
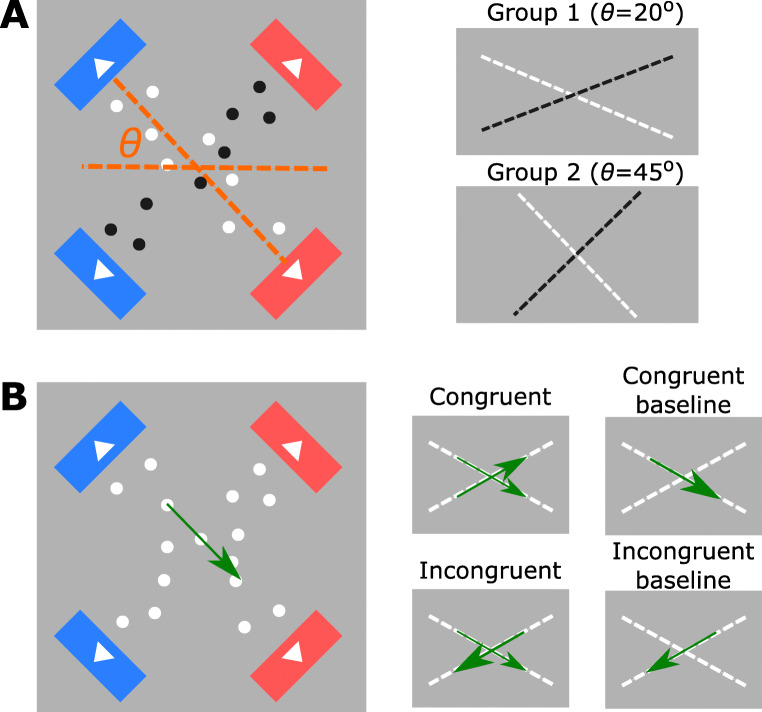
The diagram of the RDK in two rectangular apertures used in **A** the staircase procedure and **B** the main experiment. *𝜃* denotes the angle between the aperture and the horizontal plane, which was ± 20^∘^ in Group 1 and ± 45^∘^ in Group 2. During the staircase procedure, one aperture contained *black* *dots* with 0% motion coherence. In the main experiment, both apertures contained *white dots* **B**. Small diagrams in each panel represent experimental setups of each condition. *Dashed lines* represent the dots motion in respective apertures and the *colour of dots* (black or white). *Arrows* shows examples of coherent motion direction. In congruent and incongruent conditions, both apertures contained coherent motion information. In baseline conditions, only one aperture contained coherent motion information

The current study investigated these questions, using a well-established visual perceptual decision paradigm within the evidence accumulation framework. We conducted a pre-registered, carefully calibrated online experiment in two independent groups. Human participants were instructed to decide the average motion direction of random-dot kinematogram RDK from two tilted apertures (Fig. 1). Coherent dot motion was presented in both apertures, with their moving directions to be congruent (both leftwards or rightwards) or incongruent (e.g., one leftwards and the other rightwards). In corresponding baseline conditions, coherent motion was presented in a single aperture, with the other aperture containing no coherent motion. Between the two groups, we varied the angular separation of the two apertures, allowing us to evaluate the repeatability of all within-subject effects and assess the between-group effect of aperture angles on behaviour. We fitted a cognitive model, the drift-diffusion model (DDM) (Ratcliff & McKoon, 2008), to behavioural data and inferred the effects of motion congruency and sensory sources on model parameters. Furthermore, we extended a neural-mass model (Wong & Wang, 2006) to demonstrate how the observed behavioural changes can be implemented by a biologically motivated neural network. Together, using motion discrimination task based on RDK stimulus as a test platform, our study illustrated the neurocognitive mechanisms of perceptual decisions from multiple sources.

## Methods

### Participants and pre-registration

A total of 94 participants were recruited from an online recruitment portal (*Prolific*, prolific.co) and took part in the experiment online (age range 18–68 years old, median age 25 years old, 25 females, 85 right-handed). Table 1 summarises demographic features of the participants. All participants received monetary payments for their participation. Consent was obtained from all participants. We considered the recruitment from an online portal as a sample of convenience. The study was approved by the Cardiff University School of Psychology Research Ethics Committee.

**Table 1:** Statistical information about participants. NA - data not available, STD - standard deviation

Category	Value
gender	female(25), male(67), NA(1)
handness	right(85), left(6), both(2)
age (years)	median: 25, mean: 27.3, STD: 8.3
web browser type	Chrome(69), Internet Explorer(14), Firefox(4), Safari(5), NA(2)
nationality	United Kingdom(25), Poland(13), Portugal(11), United States(7), Spain(5), Italy(4), Mexico(3), Czech Republic(3), Denmark(2), Ireland(2), Hungary(2), France(2), Lithuania(1), Germany(1), Belgium(1), Sweden(1), Colombia(1), Estonia(1), Finland(1), Netherlands(1), Chile(1), Canada(1), Australia(1), South Africa(1), China(1), NA(1)

Power analyses, exclusion criteria, experiment procedures and analysis plans were pre-registered prior to data collection (https://osf.io/4dn65). A sample size of *N* > 44 provides > 90*%* power to detect a medium within-group effect (*d* = 0.5) at *α* = 0.05. We randomly assigned participants into two independent groups. Group 1 (*N* = 49) performed the perceptual decision task with two sources of visual inputs presented along *𝜃* = ± 20^∘^, and Group 2 (*N* = 45) performed the task with visual inputs presented along *𝜃* = ± 45^∘^ (see Procedure for details). This allowed us to test the hypothesis about the positive impact of the correlation of information sources on the decision accuracy.

### Apparatus

The experiment was conducted online. Experimental scripts for stimulus presentation and response collection were written in HTML with a JavaScript library jsPsych 6.0.5 (de Leeuw, 2015) and the jspsych-rdk plugin (Rajananda, Lau, & Odegaard, 2018). The online experiment was hosted on a web server Pavlovia (pavlovia.org), and participants performed the experiment in web browsers on their computers. It has been shown that online experiments in modern web browsers can serve as a suitable tool for measuring behavioural responses and reaction times with sufficient precision (de Leeuw & Motz, 2016; Semmelmann & Weigelt, 2017; Anwyl-Irvine, Dalmaijer, Hodges, & Evershed, 2020).

### Stimuli

The visual stimuli contained two independent sets of random-dot kinematograms (RDK) (Britten, Shadlen, Newsome, & Movshon, 1992; Shadlen, Britten, Newsome, & Movshon, 1996; Mazurek, Roitman, Ditterich, & Shadlen, 2003)displayed within two invisible rectangular apertures (140 pixels width, 550 pixels length) on a grey background (RGB= 128,128,128). The use of a grey background ensured that moving dots in the RDK were always visible from the background, as both white and black dots were used in the experiment. Both rectangular apertures are located at the centre of the screen, with one tilted + *𝜃* from the horizontal plane and the other tilted − *𝜃*. Hence, the two apertures formed an ‘×’ shape, with *𝜃* = 20^∘^ in Group 1 and *𝜃* = 45^∘^ in Group 2. To facilitate the integration of leftwards and rightward motion across apertures, four motion target indicators were presented at the end of the short edges of the two apertures. On each side of the screen (left or right), the two target indicators had the same colour (red or blue), and the colour assignment of those motion indicators was randomised across participants.

Each rectangular aperture contained 100 dots (i.e., 200 dots in total). Each dot had a radius of 3 pixels. We introduced coherent motion information along the long edge of each aperture (leftwards or rightwards). In each frame, a proportion of dots (namely the motion coherence) was replotted at an appropriate spatial displacement in the direction of motion (2 pixels/frame velocity), relative to their positions in the last frame, and the rest of the dots were replotted at random locations within the aperture. To minimise the impact of local motion information from individual dots, all dots were replotted at random locations after every seven frames (Rajananda et al., 2018).

The choice of two rectangular apertures serves two purposes. First, it allows us to present two independent information sources along the long edge of each aperture. Second, it allows us to present target indicators directly at the end of the short edges of the two apertures (i.e., the blue and red arrow blocks in Fig. 1). As a result, the random dot motion within each aperture leads to the percept that coherent motion direction is along the long edge of the aperture and towards one of the two short edges.

### Task and procedure

After informed consent and task instructions, the experiment included two parts: (1) a staircase procedure to identify two perceptual thresholds, and (2) the main perceptual decision-making task. In both parts, participants performed a two-alternative forced-choice (2AFC) task, deciding whether the coherent motion direction of the random-dot stimulus is leftward or rightward, either from a single information source (i.e., one aperture in Part 1, Fig. 1a) or combined from double information sources (i.e., two apertures in Part 2, Fig. 1b). Participants responded by pressing the ‘k’ key (for leftward decisions) or the ‘p’ key (for rightward decisions) on a keyboard with their right index and middle fingers. Participants were free to decide when to press the button, i.e., they performed a reaction-time version of the motion discrimination task (Ratcliff & McKoon, 2008). Each part was proceeded with a self-paced break.

#### Part 1: staircase procedure

To allow participants to familiarise with stimuli and the task, participants underwent a short practise. The practise part consisted of a single block of 32 trials. On each trial, one aperture contained black dots (RGB = 255,255,255) with 0% motion coherence, and the other aperture contained white dots (RGB = 0,0,0) at one of the four coherence levels (5, 10, 20, and 40%, eight trials of each level). Participants were instructed to pay attention only to white dots (i.e., the informative aperture) and decide the direction of coherent motion. The coherent motion direction, the order of coherence levels, and the informative aperture (i.e., the one at + *𝜃*^∘^ or the one at − *𝜃*^∘^) were randomised across trials. On each trial, the random-dot stimulus disappeared as soon as a response was made, or a maximum duration of 3500 ms was reached. The inter-trial interval was randomised between 900 and 1100 ms.

For online experiments, participants’ hardware settings and their perceptual performance could vary substantially. Therefore, we measured motion discrimination thresholds using the same visual stimulus and the 2AFC task structure as in the practise: one aperture contained black moving dots with 0% (i.e., uninformative) coherence, and the other contained white dots with motion coherence set according to the staircase routine. The direction of coherent motion was randomised across trials. At the end of each trial, visual feedback in text was presented for 500 ms to indicate whether participant’s response was correct or incorrect.

The staircase routine combined two parallel staircase procedures with fixed step sizes: one used a two-down/one-up rule and the other used a three-down/one-up rule. The two staircase procedures are independent and interleaved with each other. In both staircase procedures, the initial motion coherence was set to a supra-threshold value of 31.6%, the ‘up’ step size was 0.1 (log unit) and the ‘down’ step size was 0.074 following the recommendations from (Garcıa-Pérez, 1998; García-Pérez, 2000). Using simulations, Garcia-Perez (García-Pérez, 2000) reported the asymptotic convergence targeted by various staircase procedures with fixed step sizes, and the converged accuracy level depends on the up/down rule and the size of up/down steps. More specifically, with the down/up step size ratio of 0.74 (i.e., the value used in the current study), a two-down/one-up procedure for a forced-choice task will converge to the asymptotic threshold of 74% accuracy (hereafter referred to as the low coherence *c*_low_), and a three-down/one-up procedure will converge to the asymptotic threshold of 83% accuracy (hereafter referred to as the high coherence *c*_high_).

The convergence of a staircase procedure is an asymptotic limit, where coherence value at adjacent reversal values is sufficiently small in the limit of an infinite number of trials. In practice, a fixed number of trials is often used (Garcıa-Pérez, 1998). In the current study, each of the two staircase procedures terminated after ten staircase reversals and the corresponding threshold was calculated as the average of the motion coherence levels at the last nine reversals.

#### Part 2: perceptual decisions from double sources

Part 2 is the main experiment, in which both apertures contained white dots (Fig. 1b). Participants were instructed to attend to both apertures and decide whether the coherent motion direction of all (white) dots was leftwards or rightwards.

After task instruction and a brief practise, the main experiment comprised 432 trials, which were divided into 6 blocks of 72 trials. Participants took self-paced breaks between blocks. Decision accuracy (proportion of correct responses) was measured after every two consecutive blocks. If a participant had the accuracy lower than 60%, the experiment ended prematurely, and the dataset was discarded from further analysis.

Each block contained 50% of leftwards motion trials and 50% of rightwards motion trials. In each block, 64 main task trials from four experimental condition and eight control trials were presented. The four experimental conditions followed a 2 by 2 factorial design with two levels of combined motion coherence (high and low) and two levels of information sources (single source and double sources). Coherent motion directions, task conditions, and control trials were presented in random order across trials within each block.

In the high combined coherence conditions, trials with double information sources had the low motion coherence *c*_low_ in one aperture and *c*_high_ − *c*_low_ in the other. The coherent motion directions were *congruent* in the two apertures (i.e., both leftwards or both rightwards). We will hereafter refer to this condition as the *congruent* condition. Trials with single information source had the high motion coherence *c*_high_ in one aperture and 0% in the other , which we will refer to as the *congruent baseline* condition.

In the low combined coherence conditions, trials with double information sources had the high motion coherence *c*_high_ in one aperture and *c*_high_ − *c*_low_ in the other. Importantly, the coherent motion directions were *incongruent* (i.e., opposite) in the two apertures. We will hereafter refer to this condition as the *incongruent* condition. Trials with single information source had the low motion coherence *c*_low_ in one aperture and 0% in the other, which we will refer to as the *incongruent baseline* condition.

Therefore, for both double and single information sources, the net motion coherence was always *c*_high_ in high combined coherence conditions and *c*_low_ in low combined coherence conditions. In control trials, the motion coherence levels in two apertures were set to 60 and 0%. These easy control trials were served as attention check and excluded from subsequent data analyses.

Each trial started with a 250-ms fixation period, during which a black cross presented in the central of the screen. RDK stimuli in two apertures were then presented for a maximum period of 4000 ms, and the stimuli disappeared as soon as a choice was made. The visual stimulus was followed by an inter-trial interval randomised between 400 and 600 ms.

### Data analysis

For the staircase procedure, non-parametric tests were used to compare the high and low coherence levels (*c*_high_ and *c*_low_) and to compare between the two aperture angles (*𝜃* = 45^∘^ and *𝜃* = 20^∘^). 95% confidence intervals (CI) were obtained using bootstrap procedure with 1000 resamples of simulated distributions.

For the main experiment, we quantified response time (RT) of each trial as the latency between the RDK stimulus onset and behavioural response. To eliminate fast guesses, trials with RT faster than 250 ms were removed. Trials without a valid response were also removed. The discarded trials accounted for 0.26% of all trials. We used mixed frequentist and Bayesian ANOVAs to make group inferences on mean decision accuracy and RT, with the coherence level and the number of information source as within-subject factors. Assumptions of variance equality were checked with Levene’s test. We performed post hoc comparisons using JASP (jasp-stats.org) and used Bayes factors (*BF*_incl_, *BF*_10_) to characterise the strength of evidence (Wagenmakers et al., 2018).

### Cognitive modelling of behavioural data

We used the hierarchical Drift Diffusion Model (DDM) toolbox (Wiecki, Sofer, & Frank, 2013) to fit DDMs to individual participant’s response time distribution and decision accuracy. The hierarchical DDM assumes that the model parameters of individual participants are sampled from group-level distributions, and the Bayesian fitting procedure estimates the posterior distributions of all model parameters at both individual and group levels, given the observed data.

The basic form of the DDM contained three core parameters (Ratcliff & McKoon, 2008): (1) the drift rate *v*, (2) the decision threshold *a*, and (3) the non-decision time *T*_er_ (Fig. 5a). For each trial, the model assumes that noisy information is accumulated over time at an averaged rate of *v* and a starting point of *a*/2, until the accumulated information reaches the upper or the lower decision boundary (*a* or 0) that indicates a correct or incorrect binary response, respectively. The model prediction of RT is the sum of the duration of the accumulation process and the non-decision time, with the latter accounting for delays in sensory encoding and motor execution (Karahan, Costigan, Graham, Lawrence, & Zhang, 2019).

The effects of DDM parameters on the model’s prediction of behavioural performance have previously been documented (Ratcliff & McKoon, 2008): (1) increasing the threshold *a* leads to slower RT and higher accuracy; (2) increasing the drift rate *v* leads to faster RT and higher accuracy; and (3) increasing the non-decision time *T*_*er*_ prolongs RT but has no effect on decision accuracy. In our data, behavioural differences between conditions cannot be readily explained by a change in any single model parameter (i.e., a speed–accuracy trade-off in the incongruent condition and worsened accuracy and RT in the congruent condition). Hence, to accommodate changes in behavioural performance between conditions, we estimated four variants of the DDM with different parameter constraints. The first three variants allow two of the three parameters (*v*,*a*,*T*_er_) to vary between conditions, and the last variant allows all three parameters to vary. All parameters are allowed to vary between participants in all variants to account for inter-subject variability.

For each variant, we generated 20,000 samples from the joint posterior distribution of all model parameters by using Markov chain Monte Carlo sampling. The initial 4000 samples were discarded for the sake of obtaining stable posterior estimates (Wiecki et al., 2013). 16,000 samples has previously been shown to be sufficient for model to converge in similar tasks (Johnson, Hopwood, Cesario, & Pleskac, 2017; Szul, Bompas, Sumner, & Zhang, 2020). The convergence of the fits has been tested with a Gelman–Rubin $\hat {R}$ criterion (Gelman & Rubin, 1992). Furthermore, to improve the model’s robustness to outliers, we estimated mixture models, in that 95% of the data are explained by the DDM, and 5% of the data are expected to be outliers generated from a uniform distribution (Ratcliff & Tuerlinckx, 2002).

Model fits were assessed by comparing each model’s deviance information criterion (DIC) value (Spiegelhalter, Best, Carlin, & Van Der Linde, 2002), which takes into account both the log-likelihood function of observed data and the complexity of the model. For the best-fitting variant, we used Bayesian hypothesis testing (Gelman et al., 2013) to make inferences between conditions from the parameters’ group-level posterior distributions. For consistency, we use *p* to refer to frequentist *p* values, and *P*_*p*|*D*_ to refer to the proportion of posteriors supporting the testing hypothesis at the group level from Bayesian hypothesis testing.

### Recurrent neural mass model

We further used a neural mass model (Wong & Wang, 2006) to qualitatively demonstrate the effects of motion coherence and the number of information source on behavioural performance. The model considered here is simplified from a recurrent spiking neural network model (Wang, 2002) via the mean-field approximation. Specifically, the neural-mass model includes two simulated neural populations (i.e., accumulators), each supporting the accumulation of evidence for a direction of motion. The two simulated neuronal populations compete with each other by means of self-excitatory and mutual inhibitory connections. Each accumulator receives selective external inputs (*I*_in,L_ and *I*_in,R_) as momentary evidence supporting each alternative (e.g. leftwards vs. rightwards motion), as well as a common, non-selective background input *I*_0_ (Fig. 7a). During decision-making, two accumulators compete against each other, and the first accumulator that reaches a decision threshold renders the corresponding response. It has been shown that this biologically motivated neural mass model can explain behavioural and single-unit recording data from 2AFC perceptual decision experiments using RDK stimuli (Wong & Wang, 2006). Moreover, within a certain parameter range, the dynamics of the model can mathematically approximate that of the DDM (Wong & Wang, 2006; Bogacz et al., 2006).

Here, we extended the original neural-mass model to take into account the presence of the two information sources in the current study (for modelling details see Supplementary methods). The deterministic input currents (*I*_in,L_ and *I*_in,R_) to the two neural accumulators are given by

1 $$  \begin{cases} I_{\mathrm{in,L}}=J_{\text{ext}}[\alpha\mu(1+c_{1})+(1-\alpha)\mu(1+c_{2})]+\upbeta I_{0} ,\\ I_{\mathrm{in,R}}=J_{\text{ext}}[\alpha\mu(1-c_{1})+(1-\alpha)\mu(1-c_{2})]+\upbeta I_{0} , \end{cases} $$

where the first term is the selective input current and the second represents the non-selective background input current. *c*_1_ and *c*_2_ denote the motion coherence levels in the two independent apertures. For simplicity, hereafter we assign *c*_1_ to represent the stronger coherence between the two (|*c*_1_| > |*c*_2_|). Other parameters were set in line with previous studies (Wong & Wang, 2006; Standage, Wang, & Blohm, 2014b): *J*_ext_ = 5.2 ⋅ 10^− 4^ nA⋅Hz^− 1^ is the average synaptic coupling parameter, *I*_0_ = 0.321 nA represents the baseline of the background input current, and *μ* = 35 Hz is the baseline of input strength of the evidence.

The two scaling parameters *α* and β control to what extent task conditions affect model inputs. First, in trials with double informative sources, participants need to combine the evidence from two apertures for optimal decisions. This claim is supported by behavioural performance in the incongruent condition, in which double information sources led to lowered accuracy. If participants focused only on the dominant source, one would expect the double-source condition to have higher accuracy than the corresponding single- source condition. The parameter *α* determines how a decision- maker splits the weight of sensory evidence from two sources. *α* = 0.5 implies that a participant weight two sources equally, while 0.5 < *α* < 1 or 0 < *α* < 0.5 implies that the dominant source is weighted more or less, respectively.

Second, previous studies suggest that a change in the baseline input *I*_0_ results in speed–accuracy trade-off (Standage et al., 2014b; Heitz & Schall, 2012). Compared with the single-source condition, the double-source condition with incongruent motion directions had lower accuracy and faster RT, suggesting that participants may trade accuracy for speed in the presence of conflict information. Therefore, we assumed that the non-selective background input current is modulated by a factor of β in that condition, which changes the model dynamics and in turn affects both the accuracy and RT relative to the condition with a single information source. For other conditions, we set β = 1 such that the non-selective input is at its baseline level.

To identify the parameter regime where the neural mass model can produce qualitatively the behavioural pattern observed in the experiment, we ran model simulations with different values of *α* and β. For each parameter set, we ran 5000 simulations of each of the four experimental conditions with representative coherence levels (*c*_high_ = 20*%* and *c*_low_ = 15*%*). For example, in the incongruent condition with double information sources, *c*_1_ = 20*%* and *c*_2_ = − 5*%* for leftward motion; and *c*_1_ = − 20*%* and *c*_2_ = 5*%* for rightward motion. Mean accuracy and RT of each condition was then calculated from all simulations.

### Open data and scripts

We have made the data (https://figshare.com/articles/dataset/13567916), all analyses scripts (https://github.com/dokato/2drdk) and experimental materials (https://osf.io/5d86z/) open access (Krzemiński & Zhang, 2021).

## Results

### Behavioural results

To investigate how having multiple information sources may affect perceptual decision, two groups of participants performed a perceptual decision task, identifying the coherent motion direction (leftwards vs. rightwards) in tilted random dot kinematograms (RDKs). RDK stimuli displayed in two independent rectangular apertures, which formed a shape of ×. To further examine how the geometrical configuration of the apertures affect behavioural performance, the two apertures were presented at ± 20^∘^ for Group 1, and at ± 45^∘^ for Group 2, respectively.

**Fig. 2:**
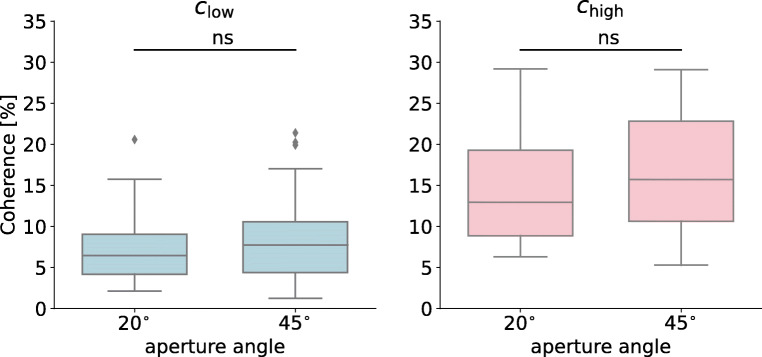
Staircase procedure results. A boxplot shows the quartiles of the data with whiskers spanning over interquartile range. *Grey dots* are outliers. Two motion coherence thresholds obtained from a parallel staircase routine: *c*_low_ from the two-down/one-up rule and *c*_high_ from the three-down/one-up rule. The final value was obtained from the last nine out of ten reversals. There were no significant (ns) differences between coherence values for the aperture angles *𝜃* = 20^∘^ and *𝜃* = 45^∘^ (Mann–Whitney *U* test)

Prior to the main experiment, each participant underwent a fixed-size, parallel staircase procedure to estimate two motion coherence thresholds, with coherent motion information randomly presented in one of the two apertures (Fig. 1a and Supplementary Fig. 1). A two-down/one-up staircase procedure was used to estimate a more difficult, or lower coherence level (*c*_low_, Fig. 2). A three-down/one-up staircase procedure was used to estimate an easier, or higher coherence level (*c*_high_, Fig. 2b). Previous research suggested that these staircase procedures lead to asymptotic convergence of 74% (for the two-down/one-up rule) and 83% (for the three-down/one-up rule) accuracy in two-alternative forced-choice tasks (Garcıa-Pérez, 1998).

Across the two participant groups, as expected, *c*_low_ was significantly smaller than *c*_high_ (*Z* = − 8.42, *p* < 0.0001, 95*%* CI = [− 8.9,− 6.3]*%*, Wilcoxon signed-rank test). We compared the coherence thresholds between the two groups, and there was no significant difference in either *c*_low_ (Fig. 2, *U*(45,49) = 983, *p* = 0.18, 95*%* CI = [− 2.7,0.9]*%*, Mann–Whitney *U* test) or *c*_high_ (Fig. 2, *U*(45,49) = 952, *p* = 0.13, 95*%* CI = [− 5.4,1.3]*%*). These results suggest that participants achieved reliable performance in the staircase procedure, and their behavioural performance was not affected by the amount of angular separation of the apertures.

In the main experiment, participants in both groups decided the combined coherent motion direction (leftwards vs. rightwards) in a 2-by-2 factorial design: either single or double apertures contained non-zero motion coherence, and the combined coherence level in the two apertures was either *c*_low_ or *c*_high_ (Fig. 1b). Critically, when both apertures contain coherent motion, two information sources are congruent in trials with a combined high coherence *c*_high_ and incongruent (i.e., with opposite motion directions) in trials with a combined low coherence *c*_low_. For simplicity, we will refer to them as the congruent and incongruent conditions, respectively, and their corresponding single-source conditions as congruent and incongruent baseline conditions (Fig. 1). This design allows us to compare behavioural performance in each double-source condition with its corresponding single-source baseline condition that had the same combined motion coherence.

We quantified participant’s performance in mean decision accuracy (proportion of correct) and RT. Each dependent measure entered a two-way mixed ANOVA with the number of information sources (single or double apertures) and congruency (i.e., congruent and baseline conditions with the combined coherence *c*_high_, or incongruent and baseline conditions with the combined coherence *c*_low_) as within-subject factors. We first focused on our primary interest: the effect of information sources, which aimed to examine whether separating information into multiple congruent or incongruent sources have the same impact on behaviour. For decision accuracy, compared with conditions of single information source, splitting motion information into two apertures always resulted in lower accuracy (marginal mean: 77.3 vs. 74.3%). This was supported by a significant main effect of the number of information sources (*F*(1,92) = 47.50, *p* < 0.001, ${\eta _{p}^{2}} = 0.34$, BF_incl_ = 1.7 ⋅ 10^4^), and there was no interaction in accuracy between the number of information sources and congruency (*F*(1,92) = 0.01, *p* = 0.92, ${\eta _{p}^{2}} < 0.001$, BF_incl_ = 0.17).

For RT, there was no main effect between single vs. double sources (1366 vs. 1354 ms; *F*(1,92) = 0.14, *p* = 0.71, ${\eta _{p}^{2}} = 0.002$, BF_incl_ = 0.12). However, the interaction between the two within-subject factors was significant (*F*(1,92) = 208.38, *p* < 0.001, ${\eta _{p}^{2}} = 0.69$, BF_incl_ = 1.2 ⋅ 10^13^), suggesting that presenting motion information in double apertures elicited different changes in response speed between congruency conditions. A post hoc test showed that compared with the congruent baseline condition, the congruent condition had slower RT (mean: 1247 vs. 1409 ms; BF_10_ = 8014.01, Bayesian *t* test). Conversely, the incongruent condition had faster RT than the incongruent baseline condition (mean: 1297 vs. 1483 ms; BF_10_ = 1.21 ⋅ 10^4^). These findings suggest that separating motion information into two spatially independent sources hinders the perceptual decision of coherent motion direction. Integrating motion information across two sources can prolong or facilitate decision speed, depending on the congruency of information in the two sources.

**Fig. 3:**
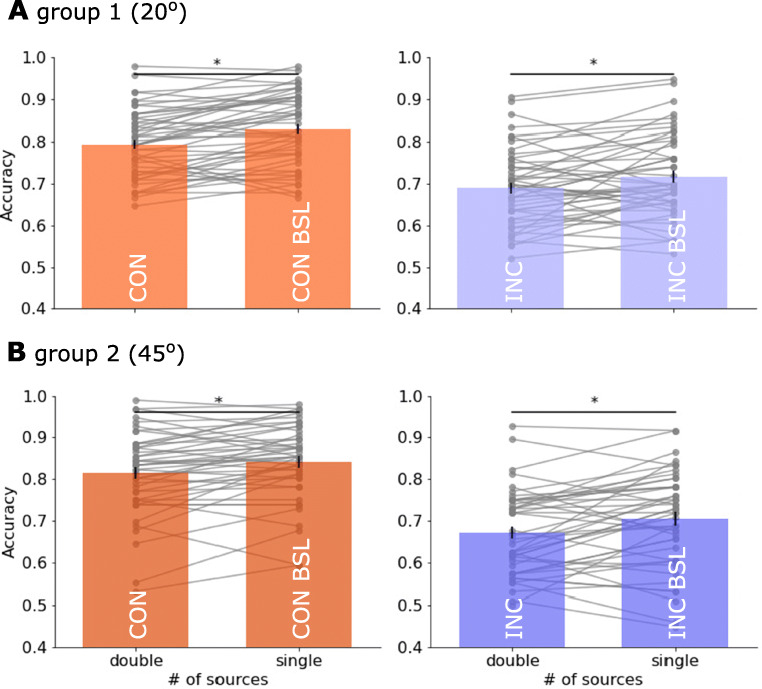
Accuracy (proportion of correct responses) in the main experiment for high (*red*) and low (*purple*) combined evidence condition. *Bars* represent the averaged accuracy in **A** Group 1 (aperture angle *𝜃* = ± 20^∘^) and **B** Group 2 (*𝜃* = ± 45^∘^). *Grey dots* represent individual participants’ accuracy. *Black horizontal line with asterisk* (*) above denotes a significance (*p* < 0.016 in all tests) of a paired *t* test between the congruent (CON) vs. congruent baseline (CON BSL), or incongruent (INC) vs. incongruent baseline (INC BSL) conditions. Each *solid grey line* links the performance between double- and single-source conditions from the same participant

**Fig. 4:**
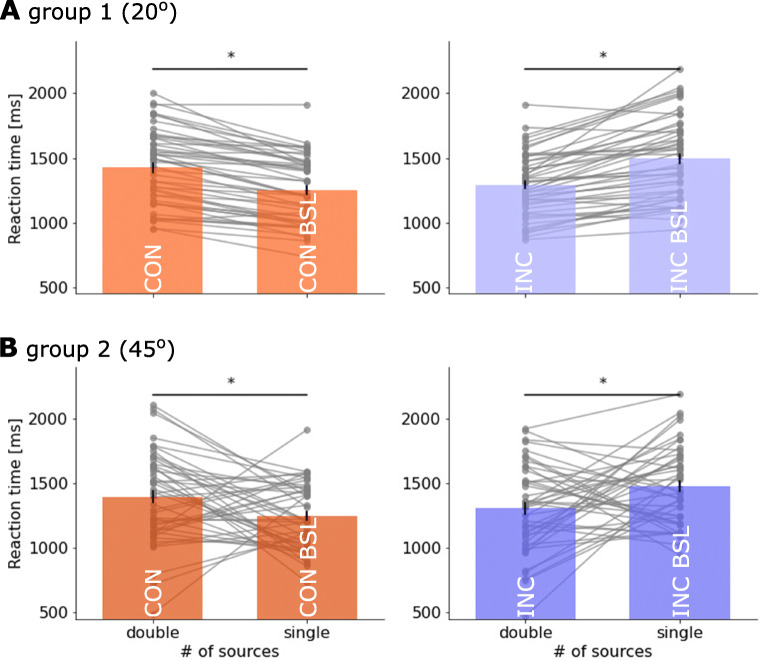
Reaction time (RT) in the main experiment for high (*red*) and low (*purple*) combined evidence condition. *Bars* represent the averaged accuracy in **A** Group 1 (aperture angle *𝜃* = ± 20^∘^) and **B** Group 2 (*𝜃* = ± 45^∘^). *Grey dots* represent individual participants’ accuracy. *Black horizontal line with asterisk* (*) above denotes a significance (*p* < 0.024 in all tests) of a paired *t* test between the congruent (CON) vs. congruent baseline (CON BSL), or incongruent (INC) vs. incongruent baseline (INC BSL) conditions. Each *solid grey line* links the performance between double- and single-source conditions from the same participant

For the factor of congruency, note that the congruent and its baseline conditions had higher combined coherence than incongruent conditions (i.e., *c*_high_ vs. *c*_low_). As expected, across both groups, the high combined coherence *c*_high_ led to better performance than the low combined coherence. This was supported by significant main effects of congruency in accuracy (Fig. 3, marginal mean: 82 vs. 70%; *F*(1,92) = 269.25, *p* < 0.001, ${\eta _{p}^{2}} = 0.745$, BF_incl_ = 2.3 ⋅ 10^55^) and RT (Fig. 4, marginal mean: 1329 vs. 1390 ms; *F*(1,92) = 53.70, *p* < 0.001, ${\eta _{p}^{2}} = 0.37$, BF_incl_ = 3.73). Hence, participants’ performance in the main task was consistent with their results from the staircase procedure, in that higher combined motion coherence led to higher decision accuracy and faster RT.

Finally, we examined the between-subject effect of angular separation of the apertures. The two groups with different angles of stimulus apertures achieved similar performance, as there was no significant group effect on behavioural performance in accuracy (75.7 vs. 75.8%; *F*(1,92) = 0.009, *p* = 0.92, ${\eta _{p}^{2}} < 0.001$, BF_incl_ = 0.29) or RT (1365 vs. 1354 ms; *F*(1,92) = 0.05, *p* = 0.30, ${\eta _{p}^{2}} < 0.001$, BF_incl_ = 0.24). Angular separation did not interact with the number of information sources (accuracy: *F*(1,92) = 0.02, *p* = 0.88, ${\eta _{p}^{2}} < 0.001$, BF_incl_ = 0.17; RT: *F*(1,92) = 0.01, *p* = 0.93, ${\eta _{p}^{2}} < 0.001$, BF_incl_ = 0.15). There was a significant interaction between angular separation and combined coherence levels in accuracy (*F*(1,92) = 4.27, *p* = 0.04, ${\eta _{p}^{2}} = 0.04$, BF_incl_ = 4.20), but not in RT (*F*(1,92) = 1.04, *p* = 0.31, ${\eta _{p}^{2}} = 0.01$, BF_incl_ = 0.19). Together, these findings suggest that behavioural performance was largely similar under different levels of angular separation.

### Cognitive modelling results

We used a hierarchical Bayesian implementation (Wiecki et al., 2013; Vandekerckhove, Tuerlinckx, & Lee, 2011)of the DDM (Ratcliff, 2002; Bogacz et al., 2006) to decompose individual participant’s accuracy and RT into model parameters that quantify latent cognitive processes. We considered four model variants, which allow the drift rate *v*, the non-decision time *T*_er_ and the decision threshold *a* to be fixed or vary between task conditions.

For each model variant, the Gelman–Rubin $\hat {R}$ convergence criterion (Gelman & Rubin, 1992) was used to assess the convergence of the last 16,000 MCMC samples from five independent Markov chains. The maximum value of the statistic from all parameters was $\hat {R} = 1.0012$, which is lower than the criterion of convergence 1.1 (Gelman & Rubin, 1992), suggesting that all parameter estimates converged after 20,000 steps.

The model variant that described the data best (i.e., the one with the lowest DIC value) allows all three parameters (*v*, *T*_er_ and *a*) to vary between conditions. To evaluate the model fit, we generated model predictions by simulations with the posterior estimates of the model parameters. There was a good agreement between the observed data and the model simulations in all conditions. Figure 6 showed group-level model simulation in comparison with empirical data (see Supplementary Figs. 2 and 3 for model fits to individual participants’ responses).

**Fig. 5:**
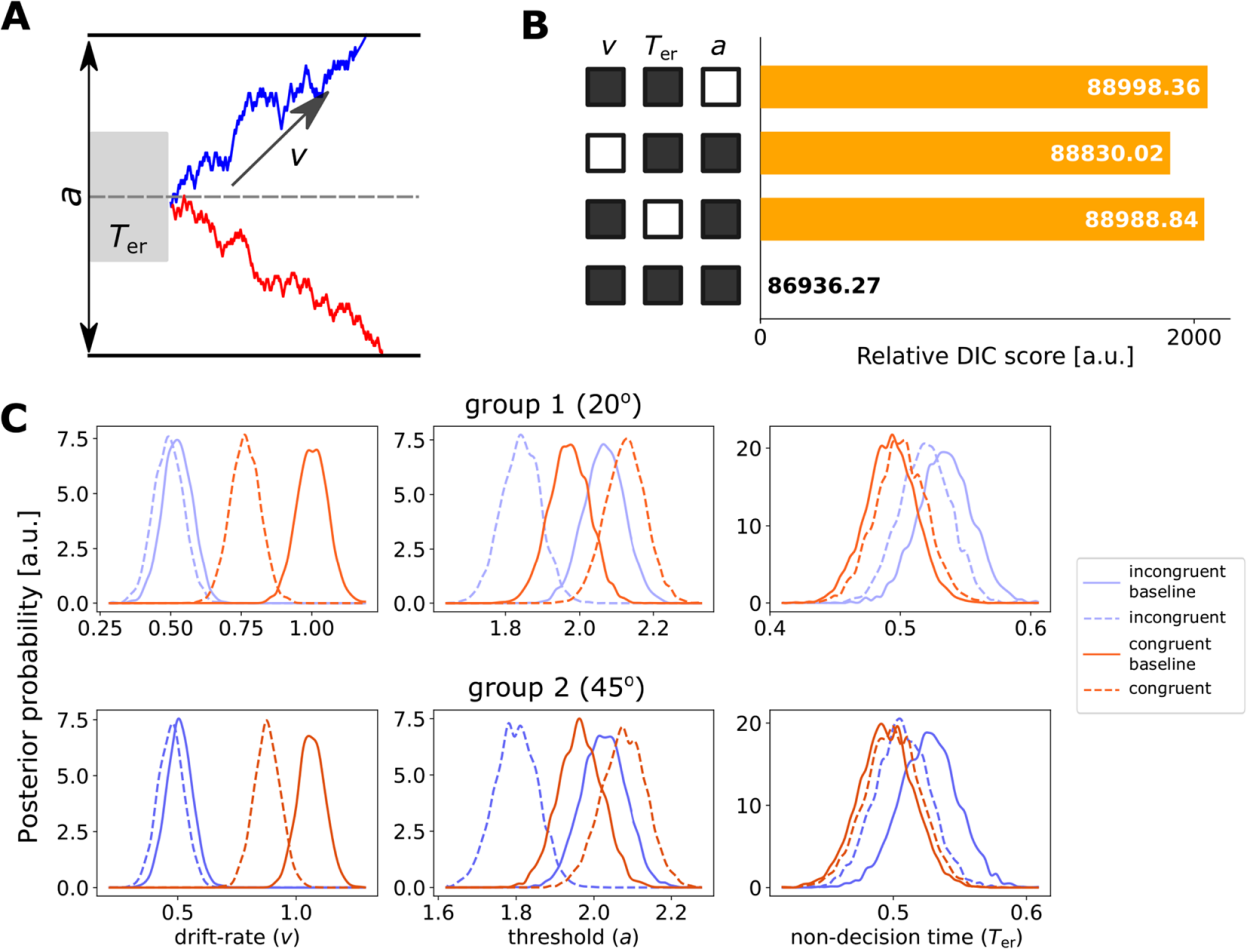
Drift-Diffusion Model (DDM) fitting results. **A** Examples of evidence accumulation trajectories depicted by the DDM. The decision threshold *a* represents the distance between the correct and incorrect decision thresholds. The drift rate *v* describes the average speed of evidence accumulation. The non-decision time *T*_er_ represents the latency of other processes not included in the evidence accumulation. The diffusion continues until the accumulated evidence reaches one of the two thresholds (*solid black lines*). If the accumulated evidence reaches the correct (*upper*) threshold (*blue trajectory*), the model predicts a correct response. Because of noise, the accumulated evidence may reach the incorrect (*lower*) threshold (*red trajectory*). **B** The deviance information criterion (DIC) value differences between the four variants of the DDM and the best fit. The *black square* indicates that the corresponding parameter can vary between the conditions, and the *white square* indicates that the parameter is invariant. The best model had variable *a*, *v*, and *T*_er_ between conditions. **C** The posterior distributions of group-level parameters of the best fit model, which is the fourth model variant in panel **B** with *a*, *v* and *T*_er_ to vary between task conditions. Each posterior distribution was obtained from 15,000 MCMC samples. *Top*: Group 1 with 20^∘^ aperture angle. *Bottom*: Group 2 with 45^∘^ aperture angle. *Red* and *purple lines* represent different congruency conditions. *Solid* and *dashed lines* represent different numbers of information sources

We then compared the posterior parameter values from the best model between experimental conditions. Inferences on model parameters allow us to examine whether making decisions with additional information sources lead to a change in the speed of evidence accumulation, the decision threshold, or both. Figure 5c shows the group-level posterior parameter estimates for the two participant groups. We used Bayesian statistics (Gelman et al., 2013; Kruschke, 2014) to quantify the proportion of parameters’ posterior distributions that did not overlap between groups and conditions (Table 2). There was no evidence to support a difference in model parameters between groups (*P*_*p*|*D*_ < 0.93 in all parameters). This concurs with the results above that the two groups did not differ in their behavioural performance.

**Table 2:** Posterior comparisons of model parameters

	*v*		*a*		*T*_er_	
	*𝜃*_1_	*𝜃*_2_	*𝜃*_1_	*𝜃*_2_	*𝜃*_1_	*𝜃*_2_
x = I1; y = I2	0.662	0.671	0.997	0.998	0.708	0.744
x = C1; y = I2	1.000	1.000	0.958	0.984	0.173	0.339
x = C2; y = I2	1.000	1.000	0.999	0.999	0.250	0.402
x = C1; y = I1	1.000	1.000	0.113	0.233	0.067	0.145
x = C2; y = I1	0.999	1.000	0.770	0.734	0.118	0.186
x = C1; y = C2	0.998	0.989	0.025	0.087	0.392	0.430

The table lists the proportion of non-overlap between two posterior parameter estimates *x* and *y*, which is equivalent to a Bayesian test of the hypothesis *P*_*p*|*D*_(*x* > *y*). Experimental conditions: I1 and I2 refer to single or double informative sources with a combined coherence of *c*_high_; C1 and C2 refer to single or double informative sources with a combined coherence of *c*_low_. The DDM model parameters: *v* drift-rate, *a* decision threshold and *T*_er_ non-decision time. Two angular distances in two groups: *𝜃*_1_ = 20^∘^, *𝜃*_2_ = 45^∘^

For the cognitive model inference, we focused on the difference between the double-aperture conditions versus their corresponding baseline condition. Compared with the congruent baseline condition, the congruent condition had a higher drift rate *v* (*P*_*p*|*D*_ = 0.006 across two groups), but there is no strong evidence to suggest a change in the decision threshold *a* (*P*_*p*|*D*_ = 0.94). In contrast, the incongruent condition had similar drift rate (*P*_*p*|*D*_ = 0.33) but a lower decision threshold (*P*_*p*|*D*_ = 0.002) than the incongruent baseline condition. We did not observe strong evidence in supporting a difference in the non-decision time (congruent vs. congruent baseline conditions: *P*_*p*|*D*_ = 0.59; incongruent vs. incongruent baseline conditions: *P*_*p*|*D*_ = 0.27). Table 2 further lists the posterior differences in model parameters between each pair of experimental conditions. Our cognitive modelling highlighted two behavioural mechanisms when information for perceptual decision is separated into two sources: a decrease in the signal-to-noise ratio of evidence accumulation (i.e., a lowered drift rate) for the congruent condition and a trade of accuracy for speed (i.e., a lowered decision threshold) for the incongruent condition. Below we investigate how these mechanisms can be implemented in a neural circuit.

**Fig. 6:**
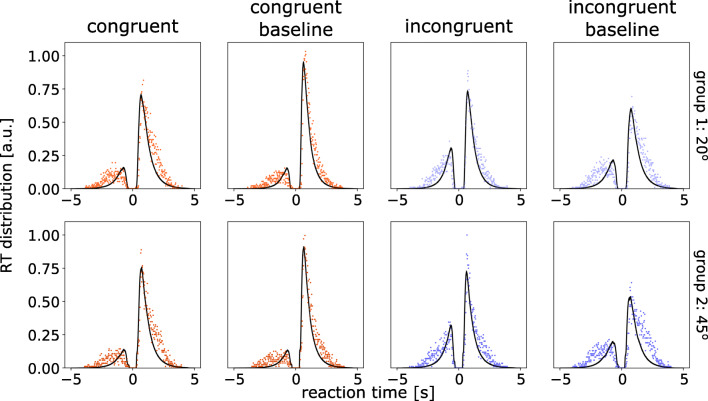
Posterior predictive response time (RT) distributions from the fitted DDM. Each panel shows normalised histogram of the observed data (red for congruent and blue for incongruent conditions) and the model predictions (*black lines*) across participants. The RT distribution of correct responses is shown along the positive horizontal axis. The RT distribution of error responses is shown along the negative horizontal axis. The posterior predictions of the model were generated by averaging 1000 simulations of the same amount of observed data. The *top row* shows results from Group 1 (with 20^∘^ angle between apertures) and the *bottom row* for Group 2 (45^∘^)

### Neural-mass modelling results

Our cognitive modelling results suggested that splitting coherent motion information into two apertures led to a decrease of drift rate in the congruent condition, and a decrease of decision threshold in the incongruent condition. How could these changes be incorporated in a biologically derived model (Wang, 2002; Standage et al., 2014b)?

**Fig. 7:**
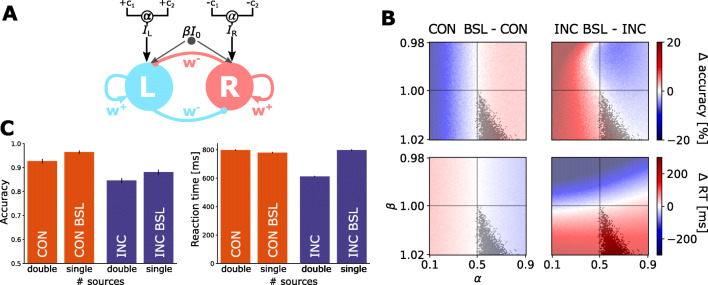
Neural-mass model simulation results. **A** The diagram of the two-state neural-mass model. *w*^+^ denotes excitatory connections, *w*^−^ denotes inhibitory connections, *α* determines how the weight of sensory evidence is split between two sources, and β modulates non-selective background input current. **B** Parameters space with difference in performance, Δ, between single and double information sources in congruent (CON BSL - CON; left) and incongruent (INC BSL - INC; right) conditions in terms of accuracy (*top row*) and reaction times (*bottom row*). The *grey zone* indicates the area where the model parameters reproduce the direction of behavioural differences observed in the experiment. *α* varied between 0.1 and 0.9, and β varied between 0.98 and 1.02. **C** Behavioural performance from model simulations with parameters *α* = 0.7 and β = 1.018. *Black vertical lines* denote standard errors from 1000 simulation runs

Building on the previous research (Standage, Blohm, & Dorris, 2014a), we hypothesised that the presence of additional information sources changes the strength of selective sensory inputs and non-selective background inputs to neural populations that implement the evidence accumulation process, which in turn leads to changes in behavioural performance. To test these hypotheses, we introduced two extensions (Fig. 7a) to a neural-mass model of perceptual decision (Wong & Wang, 2006), which implements an evidence accumulation process akin to that of the DDM (Bogacz et al., 2006). First, for conditions with double information sources, we assumed that the sensory input selective to motion coherence is a weighted sum of the two sources. The two weights (*αμ* and (1 − *α*)*μ*; see Eq. 1) sum up to the constant baseline weight *μ* that is applied to the conditions with single information source. Second, we assumed that the non-selective sensory input *I*_0_ is changed at the rate of β in the double source condition with incongruent inputs, which has been shown to be a realistic neural mechanism in modulating decision threshold (Heitz & Schall, 2012; Standage et al., 2014a).

We parametrically modulated the two scaling parameters *α* and β. For each parameter set, we simulated the extended neural-mass model 20,000 trials (5000 simulations for each experimental condition) and estimated the decision accuracy as well as mean RT. Figure 7b and c show the behavioural performance from simulations. We further identified parameter regimes that qualitatively satisfy the observed performance difference between double (both congruent and incongruent) and single information sources conditions.

Based on model simulations, in order to satisfy the pattern of behavioural performance observed in the experiment, the scaling parameter *α* on input weights needs to be larger than 0.5. Therefore, the dominant, or more informative, sensory input of the two apertures is weighted more than the other. From a modelling perspective, unbalanced weights on two sensory inputs (i.e., *α* > 0.5) imply an imperfect integration of two congruent sources, which yields lower decision accuracy than a single sensory input with the same amount of information. Recall that our cognitive model suggested a lower drift rate in the congruent condition than the congruent baseline condition. Such change in the drift rate can be mapped onto the change in the relative weighting of sensory inputs, i.e., the parameter *α* in the neural-mass model.

Furthermore, for model simulations to be consistent with the observed data, the parameter β needs to be larger than 1. This constraint implies that the incongruent condition is associated with an elevated non-selective sensory input. The higher the non-selective sensory input is, the larger activity both neural populations will have. As a result, increasing the non-selective input effectively lowers the decision threshold, because less evidence needs to be accumulated for the model to reach a decision. The change of the non-selective input in the neural-mass model serve a similar computational role as that of the decision threshold in the cognitive model: eliciting a speed–accuracy trade-off (Standage et al., 2014b; Bogacz, Wagenmakers, Forstmann, & Nieuwenhuis, 2010). In short, our neural-mass modelling offers a link between results from cognitive modelling and their potential implementations in a network circuitry.

## Discussion

The current study examined, in two independent groups, how the presence of a second source of sensory information affects the behavioural performance of perceptual decision as well as its underlying neurocognitive mechanisms. When motion directions are congruent between the two sources, decisions on the global motion direction were less accurate and slower than that in the single-source condition with the same amount of total information (i.e., combined motion coherence). In contrast, when two information sources are incongruent, decisions were less accurate but faster than that in the single-source condition. Therefore, the change in task performance depends on the congruency between multiple sources of sensory evidence.

Using a Bayesian DDM, our cognitive modelling provided novel evidence on the decision-making process with multiple information sources. First, information congruency has selective influence on different decision-making subcomponents. The congruent condition had a lower drift rate than its corresponding single-source condition (i.e., with a combined motion coherence of *c*_high_ in both). The drift rate of the DDM represents the signal-to-noise ratio of the information (Ratcliff & McKoon, 2008) and has been linked to the allocation of attention (Schmiedek, Oberauer, Wilhelm, Süß, & Wittmann, 2007). The presence of congruent information in two apertures may modulate the divided attention towards the stimuli that in turn lowers the averaged rate of evidence accumulation. Human electrophysiological data support this proposition. The EEG-evoked response potentials have been linked to the attended location in the visual decision experiments (Eimer, 1996; Luck & Hillyard, 1994). Recent studies showed that this EEG marker of selective attention modulates the rate of evidence accumulation in perceptual decision (Loughnane et al., 2016), and the dynamics of selective attention can influence evidence accumulation throughout the decision process (Rangelov & Mattingley, 2020).

Second, splitting motion information into two incongruent apertures did not vary the drift rate. Instead, there was a substantial reduction in the decision threshold, reflecting the behavioural change that participants traded accuracy for speed in this condition. The speed–accuracy trade-off (SAT) is widely observed across decision-making tasks (Wickelgren, 1977; Heitz, 2014; Beersma et al., 2003). In experiments with humans, the SAT is often induced explicitly via verbal instructions (Zhang & Bogacz, 2010) or response deadlines (Yamaguchi, Crump, & Logan, 2013). Such manipulations can efficiently switch between accuracy-seeking and speed-seeking behaviour every few trials (Mulder et al., 2013) or in consecutive trials (Forstmann et al., 2008). Modelling studies on explicit SAT demands have been consistently associated with the change of decision threshold (Palmer, Huk, & Shadlen, 2005; Ratcliff, 2006): a smaller decision threshold leads to faster and more error-prone decisions. Nevertheless, the SAT can also be triggered endogenously without explicit demands (Desender, Boldt, Verguts, & Donner, 2019). In the current study, the two apertures in the incongruent condition contained contradictory information, presenting a decision dilemma. Our results showed that in such a difficult scenario, participants adapted their decision strategy to be more speed-seeking, allowing them to complete the current decision sooner. Future research could examine this conflict avoidance bias further by changing the relative difference between multiple incongruent information sources.

Third, it is worth comparing between single- and double-source conditions which had equal motion coherence in the dominant aperture. Compared with the single-source condition with high coherence (*c*_high_ in one aperture and 0% in the other), the incongruent double-source condition (*c*_high_ in one aperture and *c*_high_ − *c*_low_ in the other) had a smaller drift rate. The congruent double-source condition had a larger drift rate than the single-source condition with low coherence. That is, introducing additional incongruent (or congruent) information led to a reduction (or increase) in the rate of evidence accumulation. These results agree with two robust behavioural effects consistently reported in the literature of visual search: the presence of distractors in hindering the search performance (Palmer, 1995), as well as the facilitating role of task-relevant information (Krummenacher, Müller, & Heller, 2002). Our findings further suggest that participants not only attended the dominant aperture, but attempted to integrate motion information across apertures to form decisions, albeit the integration of multiple information sources was not optimal, as discussed above and reported elsewhere (Wyart, Myers, & Summerfield, 2015).

Fourth, the *T*_er_ is considered as the latency external to the evidence accumulation process (Ratcliff & McKoon, 2008). Recent electrophysiological and imaging studies suggest that the *T*_er_ accounts for delays in early sensory processing (Nunez, Gosai, Vandekerckhove, & Srinivasan, 2019) or motor preparation (Karahan et al., 2019). The current study did not observe a change in the *T*_er_ between task conditions in either participant group. Hence, our results are unlikely originated from potential changes in early visual processing or motor execution in response to multiple information sources.

Based on our cognitive modelling, we proposed two extensions to a neural-mass model of decision-making (Wong & Wang, 2006). The first extension is to vary the relative weighting of sensory inputs from two independent apertures, and the second is to vary the non-selective background inputs in the incongruent double-source condition. From an exhaustive search of the parameter space, we identified the parameter regime that can qualitatively account for the observed behavioural changes in the presence of two information sources. It is worth noting that the neural-mass model is not meant to fit to experimental data, but outlines possible biological mechanisms and neural implementations that give rise to the observed behaviour.

We showed that, to accommodate experimental results, the sensory input from the dominant source needs to be weighted higher than the input from the additional source (*α* > 0.5). When this ratio becomes too high, the contribution of the additional source diminishes, resulting in the model unable to integrate information from the non-dominant source. Therefore, perceptual decisions with two information sources involve an unbalanced integration that is biased towards the more informative source.

Additionally, the non-selective background input needs to be elevated in the incongruent condition (β > 1). An increased baseline activity effectively decreases the amount of evidence required to make a decision (Standage et al., 2014a), leading to speed-seeking behaviour at the cost of less accurate decisions that was observed in the current study. Both brain imaging (Ivanoff, Branning, & Marois, 2008) and single-unit recording (Heitz & Schall, 2012) studies showed that the baseline change underlies the SAT, consistent with our model simulation results.

Interestingly, although participants were instructed to decide leftwards vs. rightwards coherent motion from two tilted apertures, the angular distance between the apertures did not affect behaviour nor DDM parameters. This may seem counterintuitive because a larger angular distance results in less coherent motion information to be projected onto the horizontal plane. Future studies could examine whether there is a significant behavioural difference at larger aperture angles because in an extreme condition of two vertical apertures (*𝜃* = ± 90^∘^), there is zero horizontal motion and the decision accuracy will be at chance. One plausible account for the lack of group difference is that participants decided the coherent motion direction with a reference of individual apertures (i.e., along their long edges), not the horizontal plane. One could validate this hypothesis by presenting multiple independent sources of motion information within a single aperture (e.g. Wendelken, Ditterich, Bunge, & Carter, 2009).

There are several limitations of this study. First, as in all online experiments, the current study faced practical constraints that could affect the millisecond-level precision of stimulus timing (Anwyl-Irvine et al., 2020). To mitigate the impact of variable testing environments between participants, we pre-registered the experiment, applied rigorous inclusion/exclusion criteria, conducted staircase procedures to calibrate stimuli for individual participants, and focused on within-subject effects in most analyses. Our study and research practises contribute to the growing trend of online psychological, or even psychophysical experiments, confirming the feasibility and reproducibility (i.e., in two independent groups) of online experiments to investigate task-specific effects in the context of perceptual decision-making (Semmelmann & Weigelt, 2017; de Leeuw & Motz, 2016).

Second, owing to the potential variability of online testing environments between participants, we designed our experiment to be completed in one testing session. Perceptual learning studies showed that behavioural performance of coherent motion discrimination improves steadily over multiple testing sessions across several days (Zhang & Rowe, 2014; Liu & Watanabe, 2012). It would be of interest to examine if repetitive training modulates the behavioural change between single and multiple information sources.

Third, for a comprehensive investigation of all possible scenarios, one could examine three conditions for each level of task difficulty: (1) a single-aperture baseline condition, (2) a double-aperture congruent condition, and (3) a double-aperture incongruent condition. The current study only included congruent conditions in high combined coherence trials and incongruent conditions in low combined coherence. This was primarily due to the constraint of limited testing time in online experiments. Furthermore, the high combined coherence was set to the threshold of 83% accuracy (i.e., staircase procedure in Part 1). Having an incongruent, high combined coherence condition in our current design would lead to strong coherent motion in the dominant aperture (e.g., *c*_high_ + *c*_low_), which may cause a ceiling effect.

Fourth, the current study aimed to use the motion discrimination task as a test platform to examine the general scenario of perceptual decision-making from multiple information sources, capitalising on existing literature and established experimental procedures (Shaw, 1982; Cassey, Evens, Bogacz, Marshall, & Ludwig, 2013). Due to our specific task settings, our conclusions may not be generalizable to broader scenarios of decision-making with other types of information. Further research should examine whether the effect of multi-source congruency on decision performance also exists in other paradigms.

In conclusion, in perceptual decisions on coherent motion direction, separating motion information into two independent sources lowered the decision accuracy. Our cognitive and neural-mass modelling showed two selective neurocognitive mechanisms that may underlie the behavioural effect, a change in the signal-to-noise ratio of the accumulation process and the speed–accuracy trade-off, depending on the congruency of multiple sensory sources. Overall, our findings combine experimental work with two levels of computational modelling, supporting potential neurocognitive mechanisms underlying the behavioural changes in decisions with multiple information sources.

## Electronic supplementary material

Below is the link to the electronic supplementary material.

(PDF 2.65 MB)
