# Correction to: Imperfect integration: Congruency between multiple sensory sources modulates decision-making processes

**DOI:** 10.3758/s13414-022-02502-6

**Published:** 2022-05-18

**Authors:** Dominik Krzemiński, Jiaxiang Zhang

**Affiliations:** 1 School of Psychology, Cardiff University Brain Research Imaging Centre, Cardiff University, Cardiff, UK; 2 Department of Zoology, University of Cambridge, Cambridge, UK

**Correction to: Attention, Perception, & Psychophysics**

10.3758/s13414-021-02434-7

There is an error in the author affiliation citation numbers both in the PDF and the HTML versions of the article.

It should be:

- Dominik Krzemiński 1, 2
- Jiaxiang Zhang 1
- Dominik Krzemiński should be affiliated both at Cardiff and Cambridge, Jiaxiang Zhang only Cardiff.

