# Supplementary material for: Imperfect integration: Congruency between multiple sensory sources modulates decision-making processes
Source: Atten Percept Psychophys. 2022 Apr 22;84(5):1566–82. doi: 10.3758/s13414-021-02434-7 (PMC9232470; doi:10.3758/s13414-021-02434-7)
Supplement: Supplementary file 1 — (PDF 2.65 MB) [file 13414_2021_2434_MOESM1_ESM.pdf]

Imperfect integration: congruency between multiple sensory sources modulates decision-making processes

Supplementary Material

Dominik Krzemiński, Jiaxiang Zhang

Supplementary methods

The neural-mass model of perceptual decision

We used the two-state neural-mass model (Wong and Wang, 2006) in the following form:

$$\frac{dS_{\mathrm{i}}}{dt} = -\frac{S_{\mathrm{i}}}{\tau_S} + (1 - S_{\mathrm{i}})\gamma r(I_{\mathrm{syn,i}}) \ , \tag{1}$$

$$r(I_{\mathrm{syn,i}}) = \frac{aI_{\mathrm{syn,i}} - b}{1 - \exp(-d(aI_{\mathrm{syn,i}} - b))} \ , \tag{2}$$

$$\begin{cases} I_{\mathrm{syn,L}} = & J_{\mathrm{L,L}}S_{\mathrm{L}} + J_{\mathrm{L,R}}S_{\mathrm{R}} + I_{in,L} + I_{\eta,\mathrm{L}} \\ I_{\mathrm{syn,R}} = & J_{\mathrm{R,R}}S_{\mathrm{R}} + J_{\mathrm{R,L}}S_{\mathrm{L}} + I_{in,R} + I_{\eta,\mathrm{R}} \ , \end{cases} \tag{3}$$

$$\tau_\eta \frac{dI_{\eta,\mathrm{i}}}{dt} = -I_{\eta,\mathrm{i}} + \eta_t\sqrt{\tau_\eta}\sigma_\eta \ , \tag{4}$$

where the index $i = L, R$ refers to two neural populations selective for leftwards and rightwards choices. The state variable S describes the NMDA gating variable (fraction of open gates). It can be shown that S has a bijection mapping on pre-synaptic firing rates (Wong and Wang, 2006). r describes the population firing rate function that depends on the synaptic input current $I_{\mathrm{syn,i}}$. The following values of the parameters were used: $a = 270(\mathrm{VnC})^{-1}$,

$b = 108$ Hz, $d = 0.154$ s, $\gamma = 0.641 \cdot 10^{-3}$, $\tau_S = 100$ ms.

The synaptic input current $I_{syn,i}$ combines recurrent inputs, mutual inputs, external inputs that relate to sensory information ($I_{in,i}$, Equation 1 in the main text) and the noise current $I_{\eta,\mathrm{i}}$. The following symmetric synaptic coupling parameters were used: $J_{\mathrm{A,A}} = J_{\mathrm{B,B}} = 0.2601$ nA and $J_{\mathrm{A,B}} = J_{\mathrm{B,A}} = 0.0497$ nA, $J_{\mathrm{ext}} = 5.2 \cdot 10^{-4}$ nA·Hz^{-1}.

The noise current $I_{\eta,i}$ is integrated with $\tau_\eta = 2$ ms (time decay of AMPA receptor activation) and random variable sample from Normal distribution η. The variance of the noise factor is kept constant $\sigma_\eta = 0.01972$.

For simulations, we use standard Euler's integration method with a time step of 1 ms.

Supplementary figures

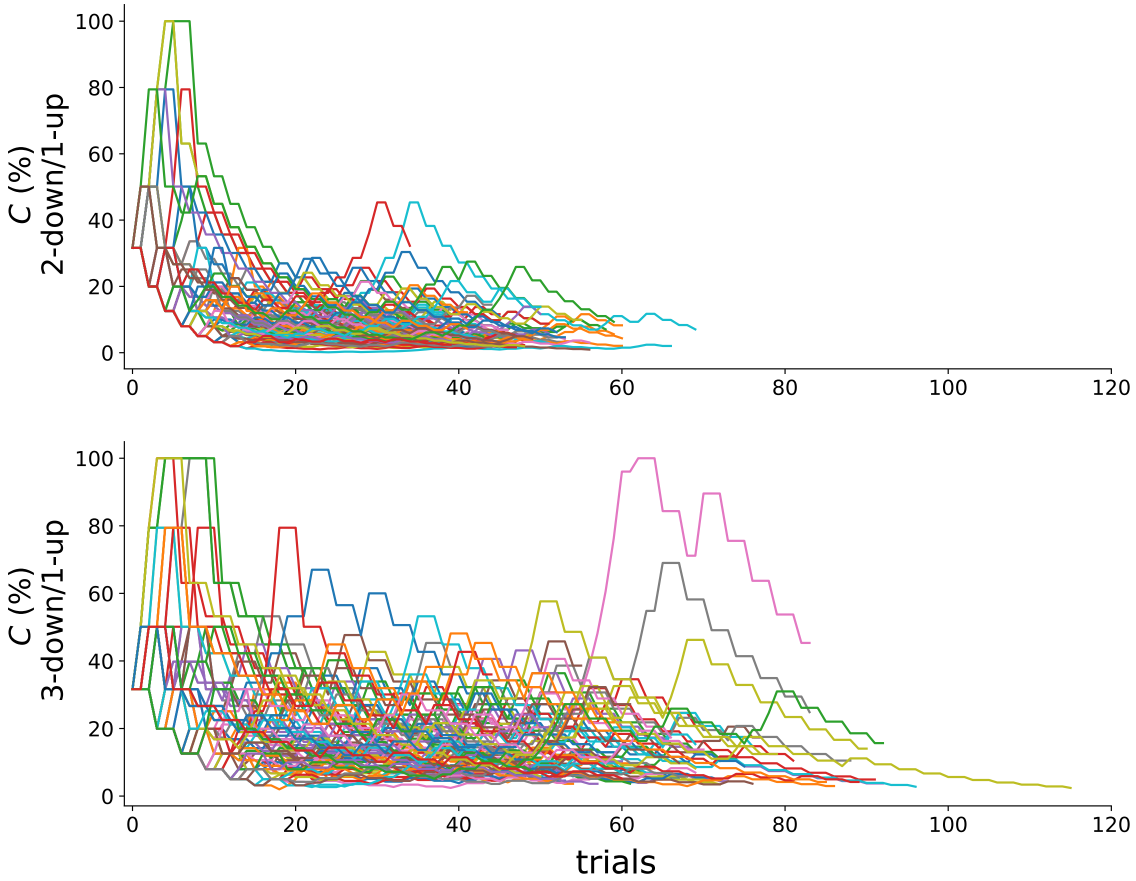

Supplementary Figure 1: Steps of the staircase procedure for the two staircase rules (top: two-up/one-down rule; bottom: three-up/one-down rule). Each line represents one participant.

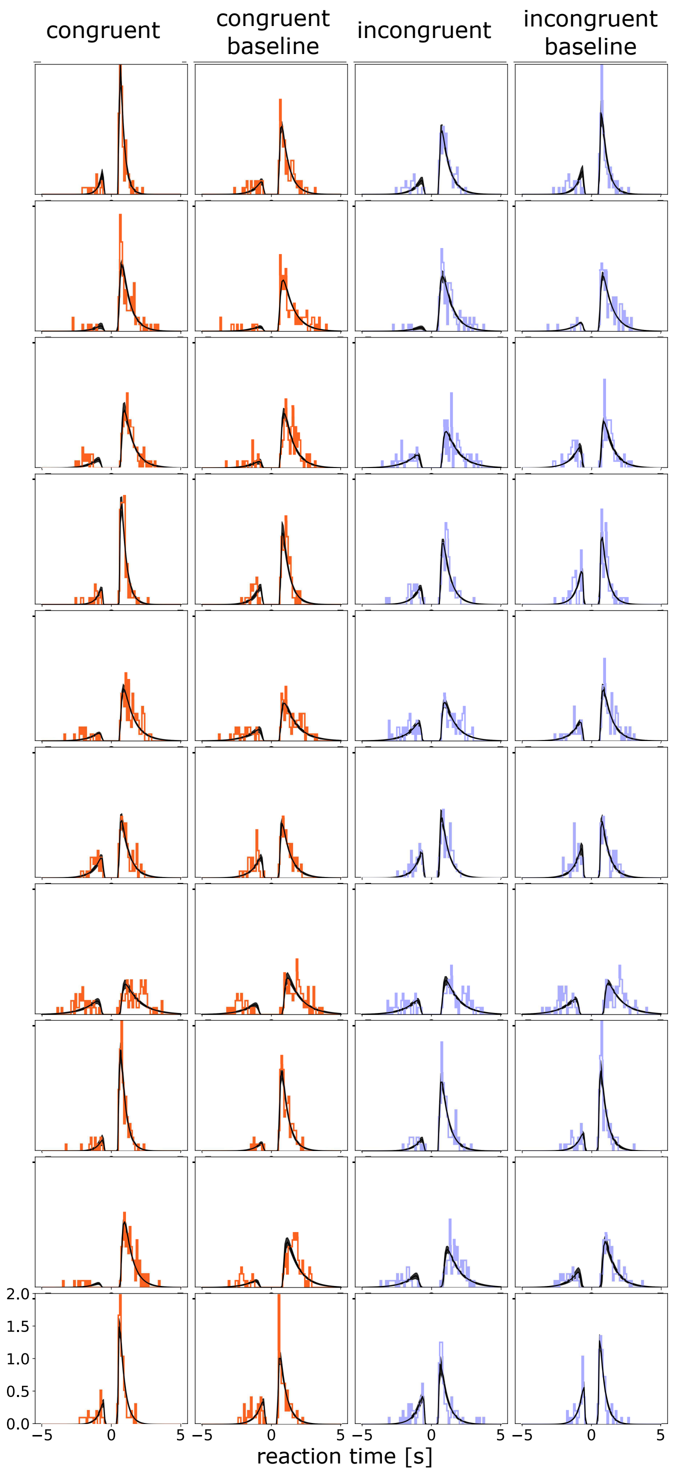

Supplementary Figure 2: Posterior predictive data distributions of 10 participants in Group 1 ($\theta = \pm 20°$). Each row shows data distributions (histograms) as well as Posterior model predictions (black lines) from the best fitted model from one of ten representative participants. The distributions along the positive x-axis indicate normalised correct response times, and the distributions along the negative x-axis indicate normalised error response times. Model predictions was generated in the same procedure as in Figure 6.

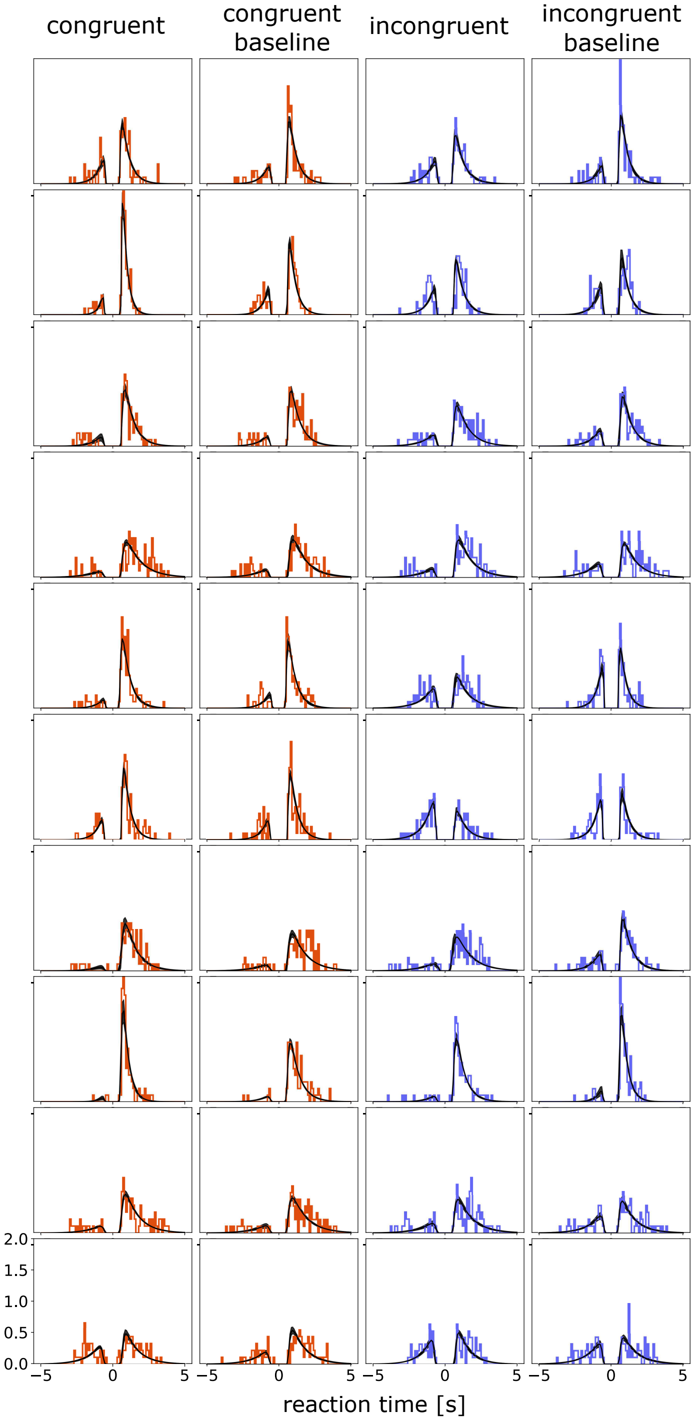

Supplementary Figure 3: Posterior predictive data distributions of 10 participants in Group 2 ($\theta = \pm 45°$). Each row shows data distributions (histograms) as well as Posterior model predictions (black lines) from the best fitted model from one of ten representative participants. The distributions along the positive x-axis indicate normalised correct response times, and the distributions along the negative x-axis indicate normalised error response times. Model predictions was generated in the same procedure as in Figure 6.

References

Wong KF and Wang XJ (2006). A recurrent network mechanism of time integration in perceptual decisions. *Journal of Neuroscience*, 26(4):1314–1328.
